# The ABC of a Simple Method for Pulmonary Vein Angiography

**DOI:** 10.1016/s0972-6292(16)30717-3

**Published:** 2014-01-01

**Authors:** Guram Imnadze, Wolfgang Kranig, Rainer Grove, Endrik Wolff, Joachim Thale

**Affiliations:** Schuechtermann Clinic, Bad Rothenfelde, Germany

**Keywords:** Pulmonary Vein Angiography

## Abstract

Catheter-directed intervention to treat atrial fibrillation (AF) is becoming widely accepted procedure in current clinical practice. For assessment of pulmonary vein (PV) anatomy, angiography of left atrium (LA) and/or PV is often performed. We present a new, simple angiographic method for PVs and LA opacification using SL1 sheath. Total of 100 patients in our clinic underwent this procedure. In all of the cases good angiographic results were achieved. No immediate or late complications related to this procedure were observed.

## Introduction

Today catheter-based AF therapy is becoming daily routine in electrophysiology (EP) laboratories all over the world. In US and Europe, more than 100000 AF ablation procedures are performed per year [1]. This procedure includes PV angiography early after transseptal puncture. Many medical centers in the world are working without modern navigation systems which obviously simplified the anatomical catheter orientation [2], so for precise localization of PV ostium an angiography is often being performed. Moreover, relatively new method of AF treatment with cryoballoon s mainly guided by PV angiography and detailed visualization of anatomy of PV side branches is recommended for inferior PVs isolation [3].

There have been several methods of PV angiography described earlier such as: selective PV angiography with the multipurpose catheter, LA angiography with pigtail catheter and injector, rotation angiography with rapid ventricular pacing and other [4]. All of those methods require additional catheter or software. We proposed a novel simple, fast and safe method of PV angiography, which does not require any additional catheter or device/interface, and provides an operator with an excellent visualization of all PV's, LA and left atrial appendage (LAA). 

## Material and Methods

From January till December of 2011, PV angiography with this method was performed in 100 patients, with median age - 59 years (35-74 years), diagnosed with paroxysmal (40) orpersistent (60) AF who were undergoing the PV ablation with conventional (65) or cryoballoon (35) technique. The mean LA size was 44 mm (30-54 mm). Thirty-five percent of our patients were female.

**Figure 1:**
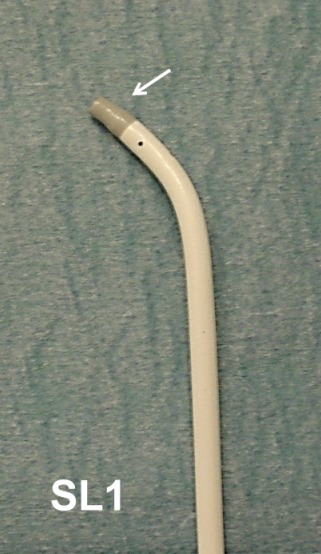
SL1 long Sheath with atraumatic tip (arrow)

After positioning the catheters (4 poles in coronary Sinus CS, 10 poles deflectable in RV) through femoral access (left 6F and 7F, right 8F) we performed transseptal puncture with traditional approach [5]. SL1 long sheath with atraumatic tip (SWARTZ St. Jude Medical) (Figure 1) and Brokenbrough transseptal needle (St. Jude Medical) were used. 34" Guiding Wire followed by the sheath placed into the LSPV (staring position). Blood was aspirated and sheath was flushed with normal saline.

**Figure 2:**
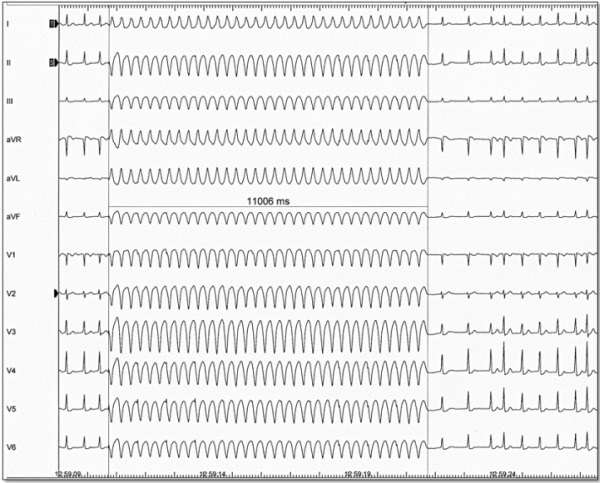
Rapid pacing from right ventricle with 300 ms in patient with AF. Duration of pacing is 11 seconds.

The angiography procedure was performed during rapid pacing (300-320 ms) from RV (in Cryoballoon technique) (Figure 2) or from LV [in radiofrequency ablation (RFA)].The Angiograms were performed with biplane cardiovascular X-ray system (Allura Xper FD10/10, Philips) in LAO 60 degree and RAO 10 degree views. AP projection is not possible with the second plane at 60 degree and less. For contrast injection 50 ml syringe was used. The entire angiography procedure was performed in the consecutive steps:

### Step A

Starts with 5th or 6th beat of ventricular pacing. X-ray and injection initiated simultaneously (right hand - bolus injection). This step comes to the end with opacification of LSPV. (Figure 3A)

### Step B

Starts after LSPV opacification completed. The sheath should be pulled back (1-2cm) with the left hand in order to reach LIPV (sometimes also LAA) and opacification (right hand - bolus injection) performed. (Figure 3B)

### Step C

As Step B completed, the sheath rotated clockwise (120-180º) with the left hand. The right hand injects the contrast bolus into the RSPV with slow but steady rise frequently opacifying RIPV at the same time. (Figure 3C)

In case the LAA is not visualized during above described three steps, the sheath is to be turned again clockwise (120-180º) and the rest of contrast injected with slow but steady rise.

An "optimal result" was defined as the angiographic views in which all PV`s including the first side branches were opacified. A "sub-optimal result" was defined as the angiographic views in which some of the PV side branches were not opacified, but ostia of all PV`s were clearly visible.

## Results

The PV-Angiography was performed without any complications in all patients. The mean contrast volume was 46.6 ml (38-50 ml). The mean time for the procedure was 9.9 sec (8-12 sec). In 89% an optimal result was reached, in 11% the result was sub-optimal. Among those with a suboptimal result, in 8% the RSPV and in 3% the LIPV side branches were not clearly visulalized, but all of those PV ostia were clearly opacified. Only one injection was required in every case, and there was no need of additional selective contrasting of any PV. In other words all the obtained angiograms were sufficient for the performance of an ablation procedure and no soft catheter or ablation catheter was required for engaging the PVs.

All of the patients with sub-optimal result had a LA diameter more than 46 mm (46-52 mm). However, even among those patients with LA diameter more than 46 mm, an optimal result was obtained in a vast majority, so the predictive value of LA diameter turned out to be not statistically significant. The position of transseptal puncture did not influence the results. Brightness of image was influenced by the Body Mass Index.

## Discussion

**Figure 4:**
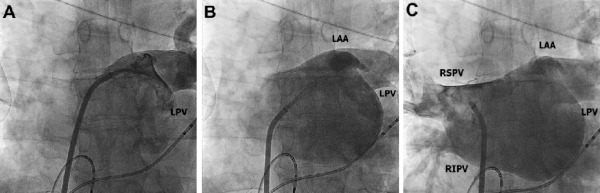
LA/PV Angiography with described method (ABC steps) in a patient with the dilated LA. In contrast to Figure 3 the image is less bright, but all of the important structures are well visualized.

Since the first publication from a Bordeaux group in 1998, which described the role of pulmonary veins in the development of AF [6], catheter ablation became the standard of care for symptomatic AF patients, who have failed at least one antiarrhythmic medication [7]. For successful AF ablation knowledge of LA and PV anatomy is crucial. The LA and PV anatomy can be assessed by selective or non-selective angiography, which can be supplemented by adenosine injection or rapid ventricular pacing for minimizing atrial emptying [8]. The new contact and noncontact mapping systems permits to assess LA and PV anatomy more precisely [9], the merging capabilities and use of intracardiac echocardiography (ICE) made the PVI procedure easier, but there is a lack of evidence translating this into improved patient outcomes [1]. Recently Yokokawa M et. al [10] concluded that preprocedural awareness of pulmonary venous and left atrial anatomy does not appear to have an effect on procedural efficiency or clinical outcomes in patients who undergo catheter ablation for AF. Additionally, the regular use of navigation systems is expensive and unfortunately not available in many countries, so fluoroscopy still remains one of the most widely applied imaging modality to guide ablation procedure. Moreover, the number of cryotherapy procedures for AF, where the detailed visualization of anatomy of PV side branches is crucial, is growing rapidly. This procedure is mostly carried out by angiography (without 3D navigation). There are many excellent articles about practicability of ICE during the cryotherapy [11], but again, it is the additional cost which limits ICE usage. We proposed a new, simple, safe and quick method of angiography, which provides a sufficient overview of the LA and the PVs, without use of additional catheters, devices or software. The above described method of angiography is a continuation of transseptal puncture and is performed with the same sheath. Around 30-50 ml contrast dye was used per procedure which usually takes less than a minute to complete (angiography itself needs 8-12 seconds). The following characteristics helped to ensure the safety of the procedure: the atraumatic tip of the SL1 transseptal sheath; the movement of catheter was limited to pulling back and rotation and the slow rise bolus injection of the contrast dye. In 2011, we performed LA/PV angiography with this technique in 100 patients. There was no complication related to this method (Patients from 2012-2013 are not included in this study but we still did not have any complications while actively utilizing this method). So we believe that the above described method is safe and can be applied in every AF ablation procedure regardless of the treatment method. The major limitation relates to dilated LA where the complete opacification may become somewhat difficult. But even in those cases, sufficient anatomical information to guide the ablation procedure can be still obtained (Figure 4).

## Conclusion

The above described method for PV angiography is proved to be a simple, safe, quick, cheap and effective tool for assessment of LA and PV anatomy for the AF ablation procedure.

## Figures and Tables

**Figure 3A:**
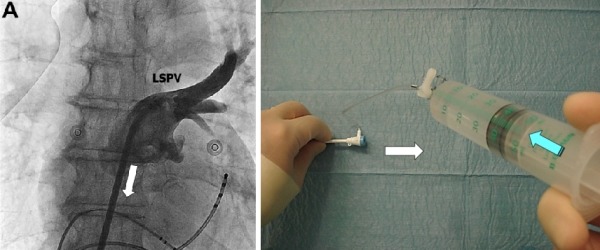
Step A: left panel shows SL1 Catheter in LSPV (starting position), The right panel shows the position of hands. White arrow describes the movement direction for the next step. Green arrow - continuous bolus injection

**Figure 3B:**
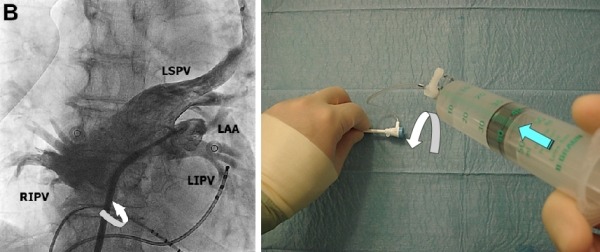
Step B: left panel shows SL1 Catheter contrasting the LIPV and LAA. The right panel shows the position of hands. White arrow describes the movement (clockwise) direction for the next step. Green arrow - continuous bolus injection

**Figure 3C:**
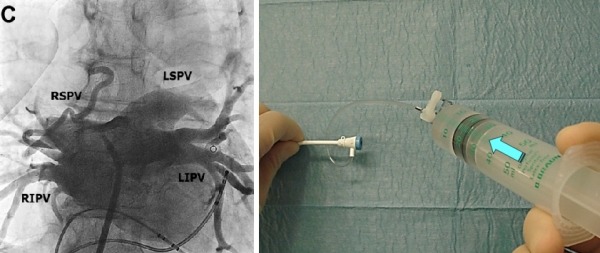
Step C: left panel shows SL1 Catheter contrasting the RSPV and RIPV. The right panel shows the position of hands. White arrow describes the movement direction for the next step. Green arrow - continuous bolus injection
